# Rheology, Spinnability, and Fiber Properties of AB-Benzimidazole Solutions in Polyphosphoric Acid

**DOI:** 10.3390/polym17172347

**Published:** 2025-08-29

**Authors:** Andrey F. Vashchenko, Ivan Y. Skvortsov, Mikhail S. Kuzin, Maria V. Mironova, Igor I. Ponomarev

**Affiliations:** 1 A.V. Topchiev Institute of Petrochemical Synthesis, Russian Academy of Sciences, Leninsky Prospekt, 29, 119991 Moscow, Russia; vaanfo@yandex.ru (A.F.V.); kuzms@ips.ac.ru (M.S.K.); mvmironova@ips.ac.ru (M.V.M.); 2 A.N. Nesmeyanov Institute of Organoelement Compounds RAS, 28 Vavilova St., 119334 Moscow, Russia; gagapon@ineos.ac.ru

**Keywords:** AB-polybenzimidazole, fiber spinning, rheology, polyphosphoric acid, drop coagulation modeling, phase separation, optical microscopy

## Abstract

This study examines the rheology and fiber formation of poly(2,5(6)-benzimidazole) (ABPBI) solutions in polyphosphoric acid (PPA) at 12.5 wt%. These solutions exhibit typical features of associative polymer systems, such as pronounced shear thinning and high elasticity. The activation energy of the viscous flow increases with the polymer concentration, reaching 29 kJ/mol at 12.5 wt%, but remains significantly lower than in phosphoric acid solutions. This indicates more efficient solvation and chain mobility in PPA. A comparison with two superbasic solvent systems further highlights the critical role of the solvent nature in flow mechanisms and associative interactions. Model coagulation experiments revealed how the non-solvent composition controls the fiber morphology and solidification. Under optimized conditions, homogeneous monolithic fibers with good mechanical performance were obtained. These findings provide new insight into the physicochemical principles of ABPBI fiber formation and establish PPA as a promising solvent for producing high-performance fibers.

## 1. Introduction

The rheological behavior and solvent interactions of rigid- and semi-rigid-chain aromatic polymers remain a subject of considerable interest in both fundamental polymer science and materials engineering. Polybenzimidazoles (PBIs) are a class of heteroaromatic polymers valued for their thermal and chemical stability, flame resistance, and durability in aggressive environments [1,2,3,4], making them suitable for demanding applications such as fireproof textiles [5,6], thermal insulation [7,8,9], heat-resistant membranes [10,11,12,13], and protective gear for firefighters [14] and aerospace personnel [15].

The most common representative, m-PBI (Celazole), is synthesized via the polycondensation of isophthalic acid and 3,3′-diaminobenzidine (DAB) in polyphosphoric acid (PPA), where the reaction mixture itself serves as the spinning solution [16,17]. However, the industrial use of m-PBI is limited by the toxicity and carcinogenicity of DAB [18].

An attractive alternative is poly(2,5(6)-benzimidazole) (ABPBI), the structurally simplest PBI composed exclusively of benzimidazole units. Due to the presence of a mobile proton and a lone electron pair on the nitrogen atom in the benzimidazole ring, ABPBI exhibits a high equilibrium moisture content—approximately 15%. Typically, such high moisture retention is only observed in natural fibers such as cotton [19], making ABPBI highly attractive for the production of flame-resistant textiles intended for use in protective clothing applications [20,21]. In addition, ABPBI demonstrates a higher degree of acid doping compared to conventional PBIs, which is particularly advantageous for applications in proton exchange membranes, where high proton conductivity is critical [22,23].

ABPBI is obtained from non-toxic and readily available 3,4-diaminobenzoic acid (DABA), which can be efficiently purified as a monophosphate salt [21]. Its high chain stiffness (Kuhn segment ~7.9 nm) and dense hydrogen bonding (interchain spacing ~2 Å) [24,25] result in superior packing, a higher density [26,27], and enhanced strength and chemical resistance [28,29]. The polymerization is tolerant to stoichiometric deviations due to the monofunctionality of DABA and allows the synthesis of high-molecular-weight ABPBI (up to 120 kg·mol^−1^) [21].

For a long time, it was believed that high-molecular-weight ABPBI could only be dissolved in aggressive solvents such as concentrated sulfuric [30] or methanesulfonic acids [27,31]. However, PPA is a condensation product of orthophosphoric acid [32] that has been shown to effectively dissolve rigid-chain polymers [30] due to its high proton-donating ability. Importantly, PPA enables the in situ combination of polymer synthesis and solution spinning, simplifying processing and reducing material losses. Furthermore, unlike sulfuric acid-based systems, phosphate-containing waste from PPA can be recycled into mineral fertilizers [33,34], offering an environmentally sustainable processing route in light of the global phosphorus scarcity and agricultural demand.

The processing of rigid-chain, non-melt-processable polymers such as ABPBI is typically achieved via wet [35,36,37,38] or dry-jet wet spinning techniques [39,40,41,42]. In wet spinning, the polymer solution is extruded directly into a coagulation bath, where fiber formation occurs through the counter-diffusion of the solvent and non-solvent. Dry-jet wet spinning introduces an air gap before coagulation, promoting a macromolecular orientation and enhancing the mechanical properties of the resulting fibers.

This study presents a comprehensive investigation into the rheology of ABPBI solutions in PPA, the mechanisms of their coagulation in non-solvents of various chemical natures for the formation of monolithic and porous fibers, and the fiber spinning processes via both wet and dry-jet wet methods. The interrelation between the solution behavior, coagulation dynamics, and fiber morphology is discussed, highlighting the fundamental aspects of structure formation in semi-rigid-chain polymer systems processed from reactive acidic media.

## 2. Materials and Methods

### 2.1. Materials

**Figure 1:**
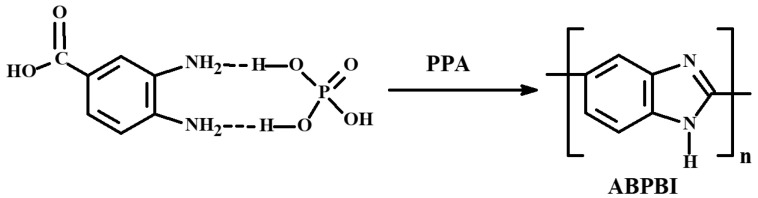
Synthesis of ABPBI from DABA-Phos.

The primary material used in this study was a 12.5 wt.% synthesis solution of ABPBI in PPA, prepared according to a previously reported procedure [21]. Briefly, 75 g of 84% PPA was loaded into a three-neck flask (Schott AG, Mainz, Germany) equipped with a mechanical stirrer RZR 2020 (Heidolph, Schwabach, Germany) and heated under Ar to 120 °C. Subsequently, 25 g (0.1 mol) of mono-phosphate salt of 3,4-diaminobenzoic acid (DABA-Phos) (INEOS RAS, Moscow, Russia) was gradually added under stirring until a homogeneous solution was obtained (approximately 2–3 h) (Figure 1). The reaction mixture was then heated to 180 °C and maintained at this temperature for 16–24 h. The resulting polymer had an intrinsic viscosity of 3.2 dL/g in phosphoric acid.

PPA (115%, Reochem, Moscow, Russia) was used as the solvent. Coagulants included bidistilled water, 40% aqueous NaOH (Reochem, Moscow, Russia), and 10% aqueous phosphoric acid (Reochem, Moscow, Russia).

Due to the presence of air bubbles in the polymer solution after synthesis, which complicates subsequent processing, degassing was performed under vacuum at 180 °C for 2 h prior to spinning.

### 2.2. Methods

#### 2.2.1. Rotational Rheology

Rheological properties of the ABPBI solutions were studied using a HAAKE MARS 60 rheometer (Thermo Fisher Scientific, Karlsruhe, Germany) in steady shear and oscillatory modes. A parallel plate geometry (20 mm diameter, glass plates) (TIPS Laboratory of Rheology, Moscow, Russia) was used. Flow curves were recorded over a shear rate range of 10^−2^ to 10^3^ s^−1^. Frequency sweeps were conducted in the linear viscoelastic region at a constant strain of 1%, in the angular frequency range of 0.6–628 rad/s.

#### 2.2.2. Coagulation Modeling and Fibers Optical Microscopy

A model drop method [43] was used to study the interaction of ABPBI solutions (with varying polymer concentrations and solvent compositions) with different coagulants. Observations were made using a laboratory polarization microscope (Polam L-213, LOMO, St. Petersburg, Russia) equipped with a ToupTek E3ISPM500 video camera (ToupTek Photonics Co., Hangzhou, China) with an optical resolution of up to 6 pixels/μm, at 25 °C.

**Figure 2:**
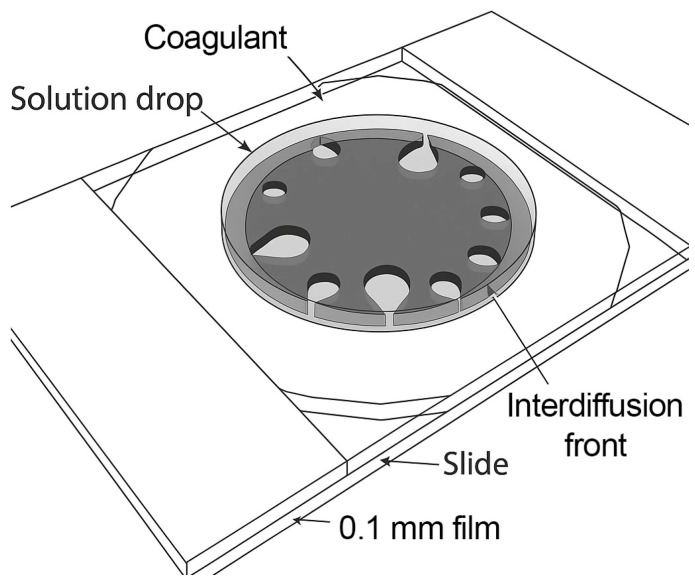
Schematic of the experimental cell used for drop coagulation modeling.

A small droplet of spinning solution (diameter ~1 mm) is placed between two glass plates separated by a 100 μm spacer and surrounded by a coagulant (Figure 2) in this method. Coagulation occurs through mutual diffusion of the solvent and the coagulant, leading to phase separation and the formation of a polymer-rich phase. This process mimics the cross-sectional morphology evolution of the fiber during coagulation in a spinning bath. The technique provides insights into the nature and kinetics of the interaction between spinning solutions and coagulants, aiding in the rational selection of coagulants without the need for extensive fiber spinning trials.

#### 2.2.3. Fiber Spinning Setup

**Figure 3:**
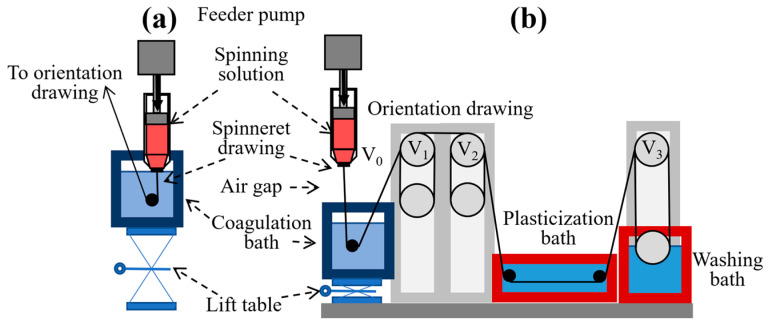
Schematic of the lab-scale fiber spinning line: (**a**) wet spinning mode and (**b**) dry-jet wet spinning mode.

Fiber spinning was conducted using a multifunctional laboratory spinning setup (TIPS Laboratory of Rheology, Moscow, Russia), comprising a Malvern RH10 capillary rheometer (Malvern Panalytical, Malvern, UK), sets of drawing rollers with adjustable speeds, heated coagulation and washing baths, and a movable coagulation bath platform. The system layout is shown in Figure 3.

The apparatus allowed collection of the fiber at different spinning stages and switching between wet and dry-jet wet spinning modes by adjusting the vertical position of the coagulation bath. Dry-jet wet spinning of ABPBI solutions in polyphosphoric acid was carried out from a custom bronze syringe (inner diameter 7 mm) (TIPS Laboratory of Rheology, Moscow, Russia) with a 3 cm air gap. The degassed polymer solution was loaded into the syringe, followed by insertion of a polyethylene terephthalate piston and attachment of a 150 μm ceramic spinneret (TIPS Laboratory of Rheology, Moscow, Russia). This assembly was installed into the rheometer in place of the standard capillary. The coagulation bath was positioned 2 cm below the spinneret. For wet spinning, the coagulation bath was raised to immerse the spinneret. Different take-up speeds of the drawing rollers were used to apply specific draw ratios.

#### 2.2.4. Mechanical Testing

Single fiber tensile testing was conducted using an Instron 1122 tensile tester (Norwood, MA, USA). Fiber samples were glued into paper frames with a gauge length of 10 mm. Fiber diameters were measured using a polarization microscope (Polam L-213, LOMO, St. Petersburg, Russia) at 25 °C equipped with a ToupTek E3ISPM500 video camera. The diameter was averaged over three points along the fiber.

Tensile testing was performed at a crosshead speed of 10 mm/min. After mounting the paper frame in pneumatic grips, the paper bridge was cut to initiate the test. All tests were conducted at 23 ± 2 °C. Reported tensile properties are averaged over at least 10 specimens from different spinning trials.

#### 2.2.5. Scanning Electron Microscopy

The surface morphology of the fibers was examined using a Phenom XL G2 scanning electron microscope (Thermo Fisher Scientific, Bleiswijk, The Netherlands) at an accelerating voltage of 15 kV.

## 3. Results and Discussion

The previously developed method for synthesizing high-molecular-weight ABPBI in polyphosphoric acid, based on a polycondensation mechanism, has demonstrated the importance of investigating the potential of PPA solutions for fiber production [18]. Preliminary studies of the molecular characteristics of ABPBI in this solvent confirmed its suitability both for fundamental research and for practical applications in film and fiber fabrication.

A key feature of the synthesis method is the use of DABA-Phos in a high-viscosity 84% PPA without mechanical stirring, with an initial monomer concentration of up to 25%. This approach yields ABPBI with molecular weights up to 94 kg/mol. The conformational analysis of ABPBI macromolecules allowed for the estimation of the Kuhn segment length (ranging from 3.3 to 5.8 nm depending on the method), confirming that ABPBI can be classified as a polymer with a moderate equilibrium chain stiffness [21].

### 3.1. Rheological Properties of ABPBI Reaction Mixtures

**Figure 4:**
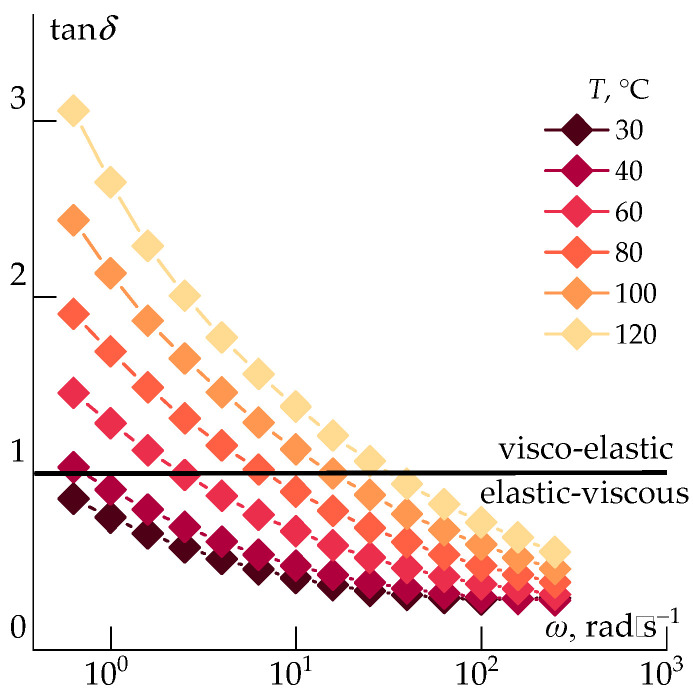
Change in viscoelastic properties of a reaction mixture upon heating.

At room temperature, the ABPBI solutions in PPA resemble rubber-like materials that can only be processed after heating, dilution, or solvent exchange. An example of the change in viscoelastic properties upon heating to 120 °C by 20 degree steps is presented in Figure 4, by the frequency dependences of the mechanical loss tangent (tan *δ*).

It is evident that the solution exhibits predominantly elastic behavior at room temperature and is unsuitable for fiber spinning. The crossover point (where *G*′ = *G*″) at 10 rad/s appears only after heating to 100 °C. At this temperature, the viscosity reaches ~10^4^ Pa·s, and the system begins to flow.

The high viscosity of reaction mixtures, combined with the corrosive and aggressive nature of PPA, limits feasible processing conditions, particularly during filtration and degassing. This also restricts fiber spinning methods and imposes stringent requirements on equipment.

Therefore, it is essential to develop approaches for controlling the rheological properties of ABPBI reaction mixtures to enable the fabrication of both monolithic and porous fibers.

**Figure 5:**
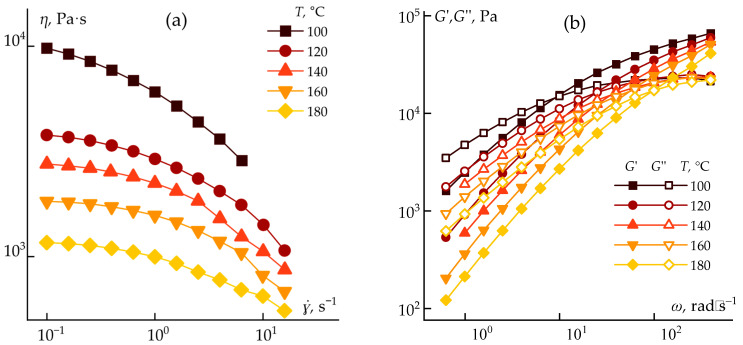
Flow curves (**a**) and storage/loss moduli (**b**) of ABPBI solutions in PPA upon heating.

Rheological measurements were performed over a range of temperatures to determine optimal processing conditions. The resulting flow curves and storage/loss modulus dependencies for temperatures from 100 to 180 °C are shown in Figure 5.

The experiments demonstrated that increasing the temperature to 180 °C results in a ~10-fold decrease in viscosity. This is accompanied by a significant shift in the crossover frequency toward higher values, enabling the use of both dry-jet wet spinning and wet spinning methods for monolithic fiber formation.

**Figure 6:**
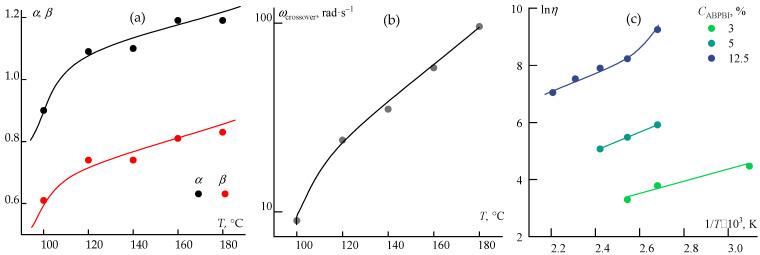
Frequency slopes (tan *α*) of *G*′ and *G*″ for a 12.5% ABPBI solution in PPA (**a**); temperature dependence of crossover frequency and (**b**) Arrhenius plot for various ABPBI solutions (**c**).

To further analyze the rheological state, the frequency dependence of the storage (*G*′ ~ *ω^α^*) and loss (*G*″ ~ *ω^β^*) moduli was studied in the terminal zone, corresponding to the longest relaxation times of the polymer chains [44]. For a Maxwellian fluid, *α* = 2 and *β* = 1 [45]. In gel-like systems, both exponents tend toward zero. Figure 6a presents the values of *α* and *β* for ABPBI solutions from 100 to 180 °C.

The influence of temperature on the crossover point, reflecting the ratio of elastic and viscous forces and corresponding to tan(*δ*) = 1, was also analyzed. This transition from viscoelastic (tan(*δ*) > 1) to viscoelastic solid (tan(*δ*) < 1) behavior is shown in Figure 6b.

A third critical parameter is the activation energy of the viscous flow, which describes the energy barrier required for layer-to-layer displacement and is expressed by the Arrhenius–Frenkel equation: *η* = *Aexp*(*E_a_/RT*), where *η* is the shear viscosity of the solution, *A* is a constant, *T* is the temperature (K), R is the gas constant, and *E_a_* is the activation energy in J/mol. The Arrhenius plots (Figure 6c) compare PPA-based systems with ABPBI solutions of different concentrations.

The analysis revealed several trends. The rheological behavior of ABPBI in PPA deviates from the classical Maxwell model, suggesting long relaxation times and partial structural organization induced by polymer–solvent interactions, which in turn affect the temperature dependence of relaxation processes. A distinct shift in the exponents (*α*, *β*) is observed between 100 and 120 °C, possibly indicating a transition in flow mechanisms. This transition is also visible in the temperature dependence of the crossover frequency and in the curvature change in the Arrhenius plot.

The optimal processing temperature for fiber spinning is above 120 °C. Despite the reduced viscosity, the solution remains significantly structured compared to conventional fiber spinning dopes [46,47].

Activation energies for the viscous flow were determined for ABPBI solutions in various solvents (Table 1).

These differences in activation energy highlight the variability in flow mechanisms depending on solvent chemistry—from well-solvated, mobile chains in PPA to entangled and networked structures in PA and superbase mixtures. This underscores the critical role of acid–base interactions and specific solvation in determining the rheological behavior. These observations reveal questions for the subsequent study of the comparative structure of these solutions using structural methods and modeling using quantum chemical modeling.

Overall, ABPBI reaction mixtures in PPA exhibit favorable rheological characteristics for direct fiber processing, with manageable viscoelasticity and relatively low activation energies. Heating above 120 °C allows for conventional spinning methods to be applied.

### 3.2. Coagulation Behavior of Reaction Mixtures

**Figure 7:**
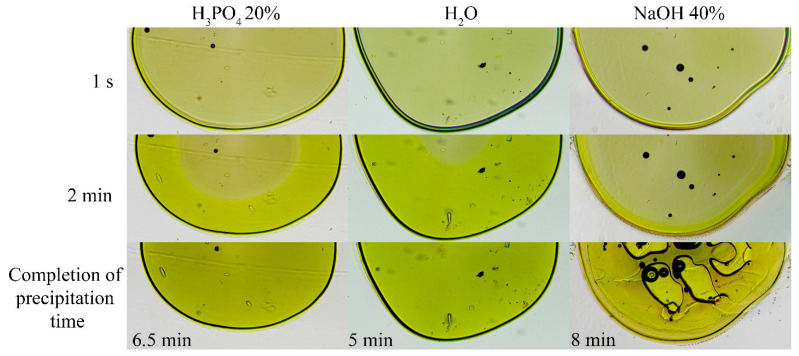
Coagulation of 12.5% ABPBI in PPA using water, diluted phosphoric acid, and concentrated alkali solution.

To investigate the influence of the coagulant nature, model experiments were conducted with ABPBI-PPA drops immersed in coagulants of different chemistries. The coagulation kinetics and resulting morphologies were evaluated. Representative results for 12.5% ABPBI solutions in acidic and basic coagulants are shown in Figure 7.

Water induces rapid coagulation compared to diluted PA but without significant changes in morphology. Coagulation by the alkali is slower and produces irregular defects. The dilution of the coagulant (as in PA) results in slight process deceleration due to the accumulation of the solvent in the coagulation medium.

These effects can be explained by differences in coagulation mechanisms. In acidic coagulants, a soft gel forms via a moving coagulation front, progressing from the droplet boundary inward. A similar mechanism occurs when water is acidified by the PPA in the drop. In contrast, basic coagulants cause immediate gelation at the polymer interface, followed by a fracture due to mass exchange and water ingress.

The experiments confirm that diluted phosphoric acid is the most technologically viable coagulant for defect-free monolithic fiber formation from ABPBI reaction mixtures. Conversely, basic coagulants promote porous structures, making them suitable for applications such as filtration materials.

### 3.3. Fiber Spinning

Fibers were produced from a 12.5 wt.% synthesis solution of ABPBI in PPA using a 0.15 mm diameter spinneret. The detailed fiber spinning conditions are summarized in Table 2.

The dry-jet wet spinning technique enabled the application of a significant temperature gradient between the polymer solution (170 °C) and the coagulation bath (25 °C), due to the presence of an air gap between the spinneret and the bath surface. However, the spinning process proved to be stable only at low or even slightly negative draw ratios (*V*_1_/*V*_0_ < 1). Increasing the draw ratio beyond unity led to frequent filament breakage.

This behavior is likely attributed to the relatively low elasticity of the hot solution upon extrusion, combined with a sudden increase in viscoelasticity upon rapid cooling in the air gap. This causes capillary instabilities and viscous thinning near the spinneret, ultimately resulting in discontinuities in the jet.

As the jet cools in the air gap, it transitions into a viscoelastic state, slowing down the coagulation upon contact with the coagulant. Due to ABPBI’s strong affinity for PPA, this results in an incomplete initial removal of acid and fibers exhibiting high elongation at the break (~100%) and moderate tensile strength (~100 MPa) with diameters around 100 μm.

During wet spinning, the hot jet immediately enters the aqueous coagulant bath. The solution coagulates rapidly due to simultaneous solvent diffusion and thermal quenching. Although the maximum temperature for the spinneret in this case is limited to 99 °C, the solution exhibits higher viscosity and elasticity. However, this immediate and sharp coagulation stabilizes the jet, allowing for higher draw ratios (up to 6.5) and the production of finer fibers.

The enhanced stretching during extrusion promotes transverse mass transfer, which facilitates the more efficient removal of residual PPA. As a result, the wet-spun fibers demonstrate higher mechanical strength and reduced residual strain.

**Figure 8:**
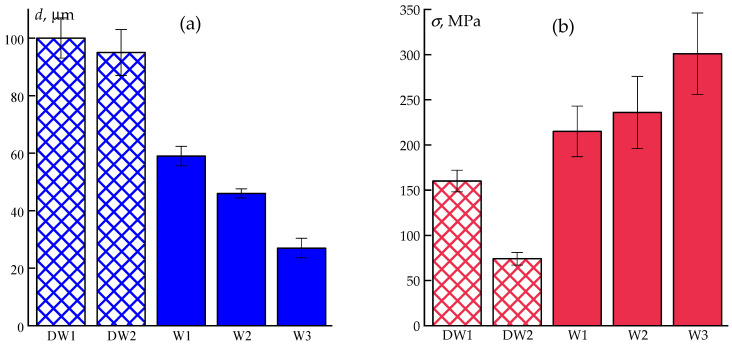

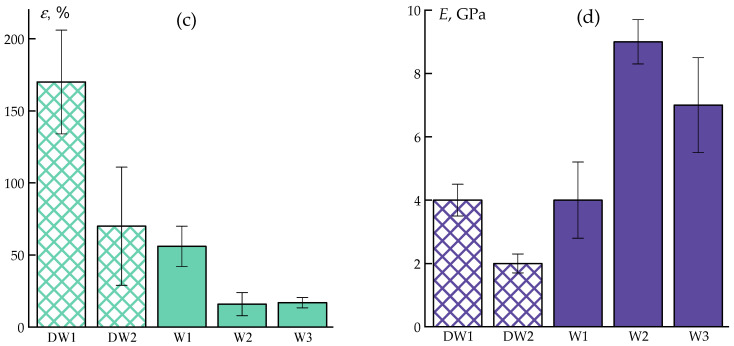
Mechanical properties of as-spun ABPBI fibers obtained via wet (W series) and dry-jet wet (DW series) spinning methods from a synthesis solution in PPA: diameters (**a**), tensile strength (**b**), elongation at break (**c**), and elastic modulus (**d**).

Data on the mechanical properties of fibers obtained in various modes using wet and dry–wet spinning methods are shown in Figure 8.

Interestingly, the experiments revealed no significant dependence of mechanical properties on the coagulation bath temperature.

### 3.4. Post-Spinning Plasticizing Draw

The most technologically viable method for enhancing the mechanical properties of monolithic fibers is additional drawing, which reduces the fiber diameter and induces a macromolecular orientation along the drawing direction. To facilitate this process, polymer plasticization within the fiber may be employed. Low-molecular-weight compounds with a high affinity for the polymer are suitable plasticizers. The high equilibrium moisture content of ABPBI suggests the potential for its plasticization by water, making hot water drawing a promising approach.

Fibers obtained using the procedures described above were subsequently subjected to plasticizing drawing in hot water (99 °C) under conditions of maximum achievable elongation. This was done to evaluate the potential of hot water to plasticize the hydrophilic ABPBI and polymer containing the residual solvent, thereby enhancing the longitudinal orientation and improving mechanical performance. Fibers produced via the dry-jet wet spinning method and containing the residual solvent were successfully drawn up to 2.5 times their original length without filament breakage, whereas the maximum draw ratio for fibers obtained by conventional wet spinning was limited to only 1.3.

**Figure 9:**
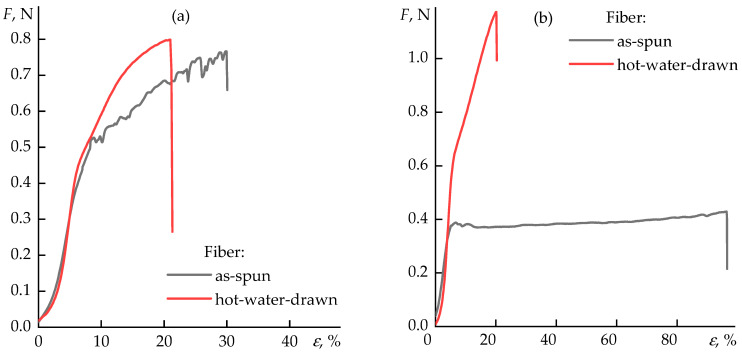
Typical stress–strain curves of as-spun fibers and hot-water-drawn fibers prepared by wet spinning (**a**) and dry-jet wet spinning (**b**).

The comparison of representative stress–strain curves for as-spun and hot-water-drawn fibers obtained by wet and dry-jet wet spinning is shown in Figure 9a,b, respectively.

The as-spun fibers exhibit a pronounced initial linear increase in stress followed by a plateau, which indicates substantial plastic deformation (up to ~150–200%). Hot water drawing leads to a polymer chain orientation, resulting in fibers with a higher modulus and the disappearance of the plastic deformation region, reflecting a high degree of molecular orientation. In the case of wet spinning, a considerable scatter in the values of elongation at break was observed, which may be attributed to the large temperature gradient between the spinneret and the coagulation bath, as well as to the non-uniform solvent removal at this stage of fiber formation.

**Figure 10:**
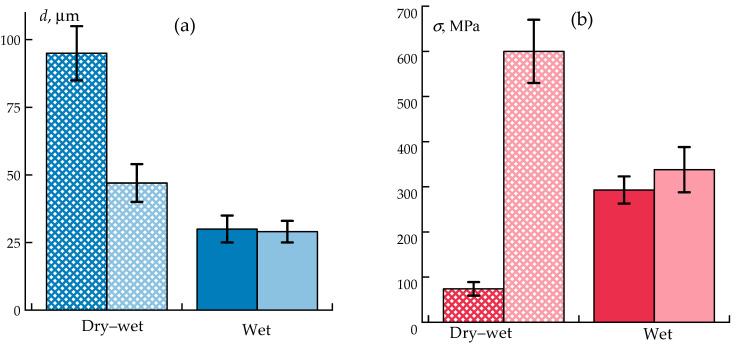

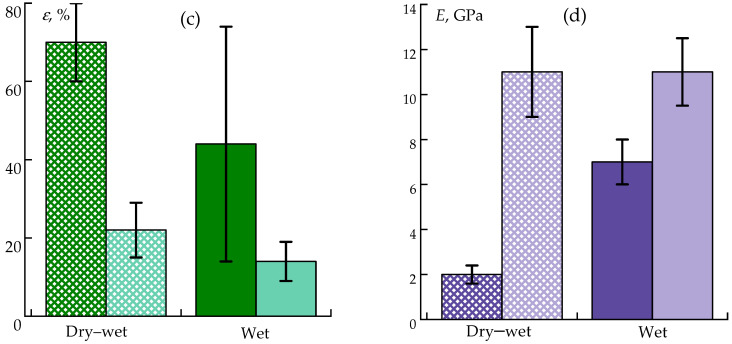
Comparison of diameter and mechanical properties of as- spun (dark color) and hot-water-drawn (light color) ABPBI fibers obtained by wet and dry-jet wet spinning: diameters (**a**), tensile strength (**b**), elongation at break (**c**), and elastic modulus (**d**).

Comparative data of the as-spun fibers and fibers after the hot water drawing are shown in Figure 10.

The results demonstrate that the post-spinning stretching of plasticized fibers is a highly efficient strategy, improving the tensile strength by up to 6-fold compared to as-spun fibers and by 2-fold compared to wet-spun, better-washed fibers. These improvements can be attributed to enhanced segmental mobility and the orientation of polymer chains facilitated by the residual solvent and water plasticization.

### 3.5. Fiber Morphology Analysis

**Figure 11:**
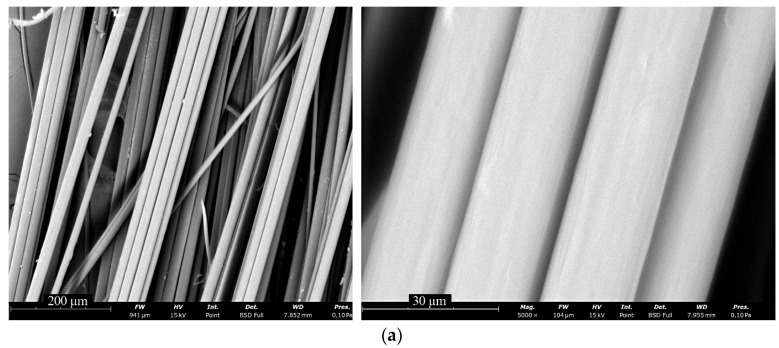

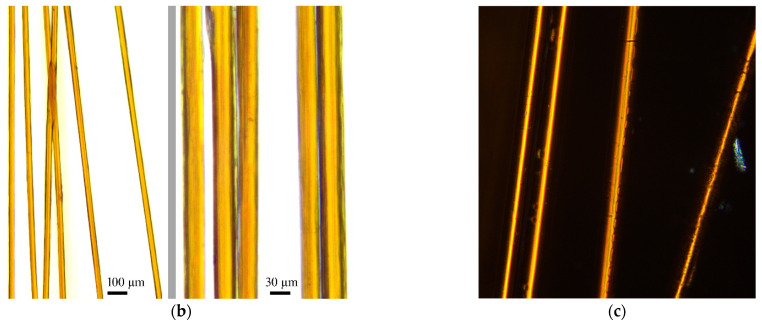
Surface morphology of ABPBI fibers observed by (**a**) SEM, (**b**) transmitted optical microscopy, and (**c**) polarized light microscopy.

Surface morphologies of the fibers were characterized using both optical and scanning electron microscopy. The findings are presented in Figure 11.

The fibers exhibited uniform diameters and smooth surfaces without vacuole-like defects, which correlates well with prior observations from droplet coagulation modeling. The fibers were optically transparent and lacked gross structural inhomogeneities. A faint fibrillar texture could be observed, consistent with an axially oriented polymer network.

Moreover, strong optical birefringence was noted, which indicates a high degree of molecular orientation along the fiber axis. This is particularly noteworthy, given that ABPBI is considered largely amorphous based on previous structural studies [48].

## 4. Conclusions

This study demonstrates the direct transformation of ABPBI synthesis solutions in polyphosphoric acid into strong, oriented fibers. The rheological analysis revealed that high-molecular-weight polymers in viscous media can be processed effectively upon heating due to the favorable polymer–solvent interactions, providing stable spinning conditions. Coagulation experiments highlighted the decisive role of the coagulant miscibility and neutralization strength in controlling the phase separation and preventing porosity. Optimized spinning and drawing yielded monolithic fibers with high mechanical strength. The presented findings offer a comprehensive understanding of the dissolution–coagulation–orientation pathway in fiber spinning from PPA and lay the foundation for the future development of direct methods from high-viscosity solutions in polyphosphoric acid.

## Figures and Tables

**Table 1:** Activation energies of viscous flow for ABPBI in different solvents.

Solvent	Concentration (%)	*E_a_* (kJ/mol)
Polyphosphoric acid	3/5/12.5	17/26/29
Orthophosphoric acid	3/5	28/40
DMSO/MeOH/KOH (10% MeOH) [48] *	3/7/9	20/35/70
DMSO/MeOH/KOH (60% MeOH) [48] *	3/7/9	20/25/30

* Detailed data are published in the work [48].

**Table 2:** Parameters of ABPBI fiber spinning from a 12.5% synthesis solution in PPA.

Sample	Method	*T_solution_*, °C	*T_coagulant_*, °C	*V*_1_/*V*_0_ ^1^
DW1	Dry–wet	170	25	0.77
DW2			25	1
W1	Wet	99	25	1.6
W2		99	70	1.7
W3		99	25	6.5

^1^ *V*_1_/*V*_0_—is the drawing ratio between the roller *V*_1_ and the linear flow rate of the solution from the die *V*_0_.

## Data Availability

The data that support the findings of this study are available from the corresponding author upon reasonable request.

## Abbreviations

The following abbreviations are used in this manuscript:

ABPBI	poly(2,5(6)-benzimidazole
PPA	polyphosphoric acid
PBIs	polybenzimidazoles
DAB	3,3′-diaminobenzidine
DABA	3,4-diaminobenzoic acid
